# Reducing the complexity of financial networks using network embeddings

**DOI:** 10.1038/s41598-020-74010-2

**Published:** 2020-10-12

**Authors:** M. Boersma, A. Maliutin, S. Sourabh, L. A. Hoogduin, D. Kandhai

**Affiliations:** 1 Computational Science Lab, University of Amsterdam, Amsterdam, The Netherlands; 2 KPMG, Amstelveen, The Netherlands; 3 KPMG Global Solutions Group, Berlin, Germany

**Keywords:** Applied mathematics, Computational science

## Abstract

Accounting scandals like Enron (2001) and Petrobas (2014) remind us that untrustworthy financial information has an adverse effect on the stability of the economy and can ultimately be a source of systemic risk. This financial information is derived from processes and their related monetary flows within a business. But as the flows are becoming larger and more complex, it becomes increasingly difficult to distill the primary processes for large amounts of transaction data. However, by extracting the primary processes we will be able to detect possible inconsistencies in the information efficiently. We use recent advances in network embedding techniques that have demonstrated promising results regarding node classification problems in domains like biology and sociology. We learned a useful continuous vector representation of the nodes in the network which can be used for the clustering task, such that the clusters represent the meaningful primary processes. The results show that we can extract the relevant primary processes which are similar to the created clusters by a financial expert. Moreover, we construct better predictive models using the flows from the extracted primary processes which can be used to detect inconsistencies. Our work will pave the way towards a more modern technology and data-driven financial audit discipline.

## Introduction

Creditors, lenders, and investors make high impact investment decisions based on financial reports on a daily basis. Therefore, it is critical that the financial reports give a true and fair view of the financial state of a company. The importance of audit is well-recognized and most countries have introduced laws that mandate the audit of financial information. As a result, the global audit market has grown to a 210 billion dollar industry by 2017^1^. Accounting scandals such as Enron (2001), Parmalat (2003), Lehman Brothers (2008), Petrobras (2014) and more recently Malaysia Development Berhad (2018) emphasize the consequences of unreliable financial information which can lead to unexpected defaults. Moreover, it can lead to economic instability and even be a potential source of systemic risk as a result of cascading effects^2–4^.

Traditional audit procedures are mostly manual in nature. Auditors’ conclusions based on these procedures are predominantly judgmental and therefore less consistent between audits. Predictive models can overcome these limitations by objectively detecting inconsistencies between monetary flows^5–10^ within the financial information. Increased objectivity will lead to higher quality audits. However, despite the surge of available financial data in the recent years, predictive models are not widely used in financial audits^11, 12^.

In order to construct predictive models for audit purposes, it is important to know the interaction between different components of the financial statements, which is referred to as the structural information of the company^8–10, 13, 14^. Chen et al.^10^ define a generic structural model whereas in our previous work^15^ we proposed a network representation that extracts the structural information from transaction data. However, this results in high-dimensional networks which are difficult to interpret. In order to facilitate the analysis of these networks, we reduced the dimensionality by grouping similar nodes into clusters using the opinion of an audit expert. It was shown that useful predictive models can be constructed from the low-dimensional representation of the network. The drawback of this methodology is that the involvement of an expert for the clustering task is inefficient, inconsistent and subjective.

The recently emerged research field of graph embedding^16–20^ enables low-dimensional representations of large-scale networks, thereby ensuring tractable visualization and analysis of the resulting networks. Domains like sociology and biology have successfully applied these techniques to perform a variety of tasks such as node classification, link prediction and clustering^21–23^. The foundation of this technique originated from statistical language modelling^24^ which learns similarities between words. The meaning of a word is implied by the context words surrounding it, words with similar context have similar meaning. Statistical language modelling infers these relationships from a large text corpus. It was later applied to networks by Perozzi who proposed DeepWalk^21^ to embed social networks. Here similarity between nodes is defined as a similar neighborhood in the network. To embed the nodes in the network similar to words in sentences, we perform a random walk on the network structure that samples possible paths. Each sampled path is used as a training set for the following prediction problem (Skip-Gram model^24^): learn a vector representation such that nodes with a similar context will have a high similarity score between their embedded vector representations. Variations of DeepWalk have been proposed to suit different types of networks or preferences, like heterogeneous networks^25^, bipartite networks^26^ and more^27, 28^.

Networks also play an important role in understanding the complexity of systems. In a recent study Flood et al.^29^ quantified the organizational complexity of a bank holding company, complexity is determined by the number of heterogeneous edges in the ownership network, i.e. an edge between two different types of nodes where the nodes represent entity types, e.g. domestic or non-domestic. In turn, in the research field of biology, understanding the function of a protein is important. For unknown proteins the network is used to predict the function of the protein through a principle called ’guilt-by-association’^30^, predicting the function through the interaction with other proteins of which the function is known. Sedina et al.^30^ show that taking the oscillating dynamics into account improves the function prediction of proteins.

Networks are a generally useful representation to address domain-specific tasks. In this article, we apply techniques from network embedding to the networks extracted from transaction data, for which the embeddings are used to cluster the nodes. These clusters form the basis for the down-stream prediction tasks for the audit that can be used to detect inconsistencies. In addition, we propose a novel adaptation of DeepWalk which will improve the performance of the clustering task in the simulated dataset. The performance of the clustering is evaluated using three real datasets annotated by an expert as a benchmark. Furthermore, we generate simulated transaction data with realistic characteristics to assess the robustness of our method. Finally, we assess the impact on the down-stream prediction task as a result of the clusters obtained from the embeddings. Our results show that we can embed the financial statements network in an effective way, and, more importantly, that these embeddings can serve as a foundation for other modern data-driven techniques that can be applied in the audit.

## Results

### The complexity of financial statements networks

**Figure 1:**
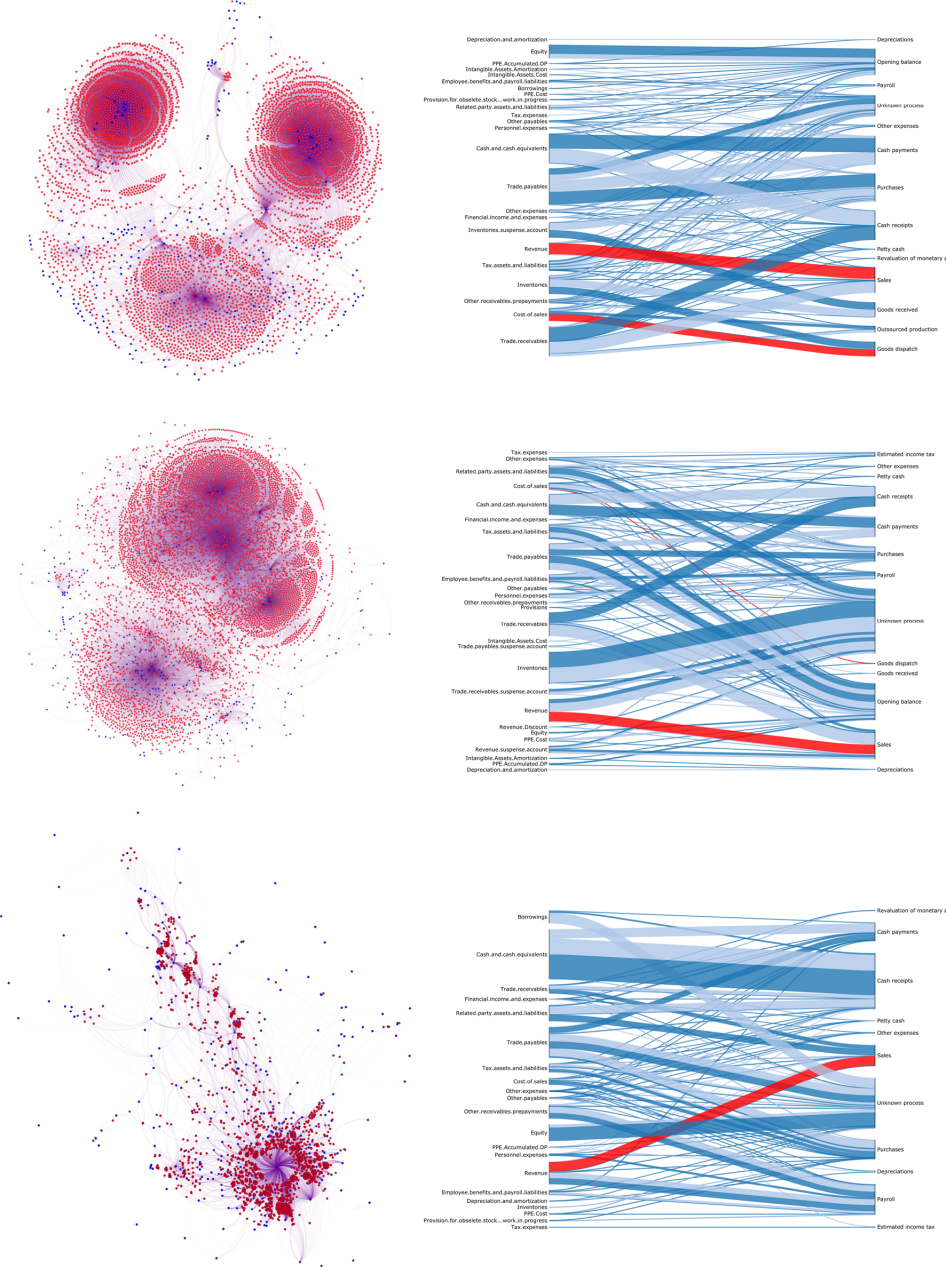
The two bipartite networks of three companies, the left side is the detailed network with financial account nodes in blue and business process nodes in red. On the right, the simplified bipartite network of the network on the left is displayed. In this network, we display on the left the financial accounts and on the right the business processes. In the simplified bipartite network the edge width represents the monetary flow. The dark blue represents outflows from financial accounts and the light blue represents inflows. The top left network is a new rendering of the same network as displayed in Boersma et al.^15^.

In this study, we first show the complexity of the network obtained from three real companies. In Fig. 1 (left) we show the complexity of this bipartite financial statements network of the three companies. The bipartite network consists of two distinct sets of nodes, a set of financial account nodes in blue and a set of business process nodes in red. These are distinct sets because there are only edges between nodes from either set. The financial account nodes represent what kind of monetary value propagates through the network, and the business process nodes describe which process drives the propagation. The networks have 4,800+, 6,800+ and 1,800+ nodes and 35,000+, 29,000+ and 6,200+ edges, respectively. In order to render the networks, we used the Fruchterman-Reingold method^31^. In all resulting networks we observe cluster formation and highly connected nodes. Even though these networks seem to show interesting structures, it is infeasible for an auditor to analyse these. Figure 1 (right) shows the simplified bipartite network, an easy to read visual representation of the monetary flows, with on the left side the financial account nodes and on the right side the business process nodes. The number of nodes is reduced by grouping similar nodes and the monetary flows are the edges between the nodes.

Before proceeding to examine the detailed network, it is important to understand how the predictive models use the network structure. Figure 1 (right) shows the high-level monetary flows that can be used to construct predictive models. In particular, we use the network to distinguish between in- and outflows, and process flows. If a latent dependency exists between flows, then these process flows can be used to construct predictive models. For example, the relationship A: between Revenue and Cost of Sales, both red highlighted flows in the network (see Fig. 1 (right)). However, these high-level networks are constructed with the help of an audit expert that grouped the nodes.

**Figure 2:**
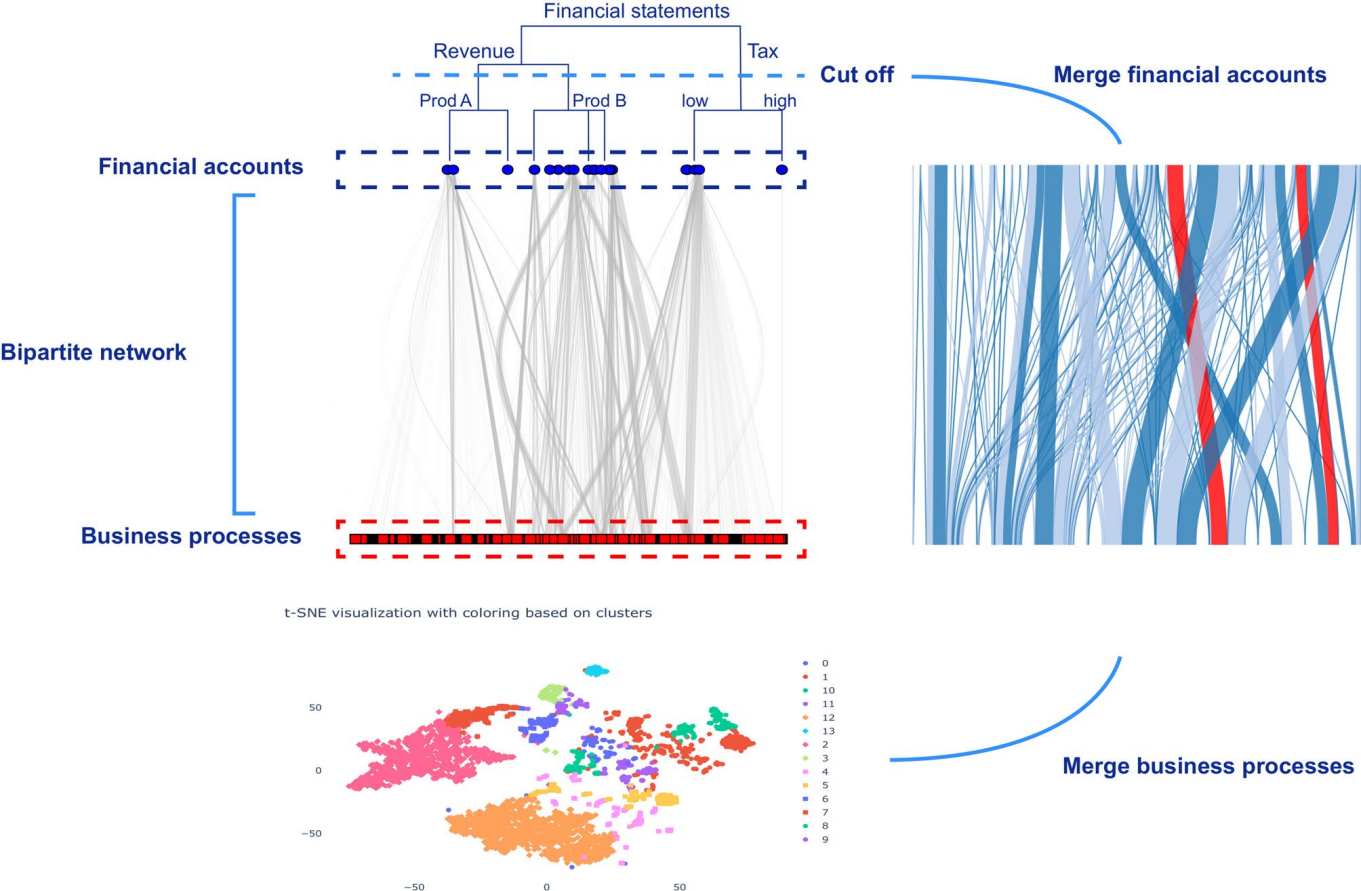
The bipartite network extracted from the transaction data. The blue nodes represent the financial accounts and the red nodes the business processes. On top of the blue nodes we show the hierarchical organization of the financial accounts up to the financial statement level. The bottom part is the t-sne visualization of the embedding values of the business process nodes which we can cluster using agglomerative clustering. The network on the right side is the resulting network after we grouped the financial account nodes according to the cut-off in the financial accounts hierarchy and the business process nodes according to the clusters found in the embedded space. On the basis of this network we construct model A, the relationship between Revenue and Cost of Sales based on the profit margin.

With regard to this node grouping task, the objective of the network embedding is to learn a low dimensional vector representation of each node in such a way that a clustering algorithm can properly group the nodes. In Fig. 2 we show the overview of our approach where on the left the detailed network is presented and, on the right, the high-level flow network. In order to construct this, we need to merge both the financial account nodes and the business process nodes. On the one hand, for the financial account nodes we use the known financial hierarchy to merge the nodes, i.e., the hierarchy that tells us how financial account values are aggregated to the totals presented in the financial statements (see Fig. 2 financial hierarchy). On the other hand, merging business processes is less straightforward because no such hierarchical structure is known. Therefore, we propose to use the network embedding to merge the nodes into clusters. We use the agglomerative clustering method that provides us with the hierarchical organization of these clusters similar to the financial hierarchy.

### Clustering business processes with network embedding

We find that the clusters detected represent to a high degree the core processes of a company as indicated by the expert auditor.

On the question to which degree the clusters are similar to the expert’s clusters we use a quantitative v-score^32^ measure between 0 and 1 (see “Methods” section). This score is the harmonic mean between the homogeneity of the clusters, i.e. does one cluster consist of a single label class, and completeness, i.e. does one label class belong to a single cluster. Ideally, we would have one cluster for each class, resulting in a perfect v-score of 1.

#### Core processes

As explained earlier, we compare our clusters with the expert’s clusters. Therefore, the expert identified the following core processes in the dataset of company A: Cash receipts, Purchase, Cash payments, Sales, Goods received, Goods dispatch and Outsourced production. These processes account for 95% of the monetary transfer, the remaining business processes are assigned to the Unclassified category. Boersma et al.^15^ show that this category could be quite sizable. In addition, the expert identified processes such that we have a total of 14 processes. For company B and C the expert used a similar approach to categorize the nodes into core processes. For company B we have 13 clusters and for Company C we have 11 clusters. For both we see that a larger proportion of the nodes fall within the Unclassified category.

**Figure 3:**
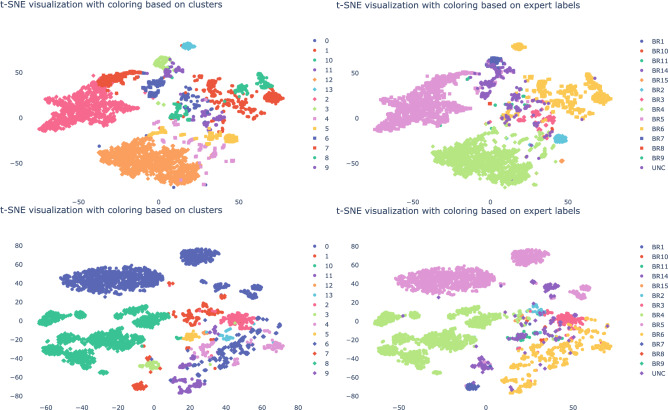
The t-sne visualization for company A obtained with finWalk (top) and DeepWalk (bottom), with on the left the clusters as indicated by the agglomerative clustering algorithm and on the right the expert’s labels.

**Figure 4:**
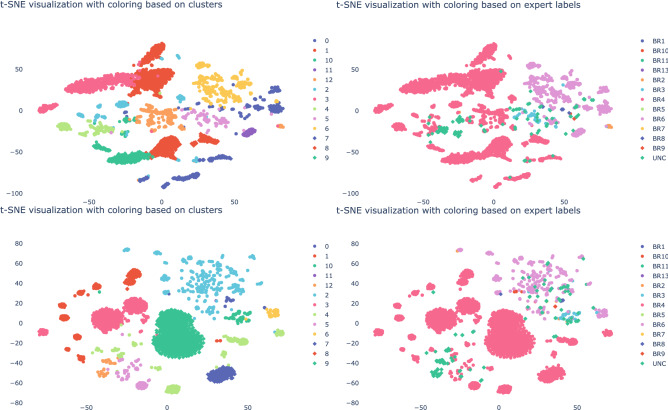
Here we show the t-sne visualization for company B obtained with finWalk (top) and DeepWalk (bottom), with on the left the clusters as indicated by the agglomerative clustering algorithm and on the right the expert’s labels.

**Figure 5:**
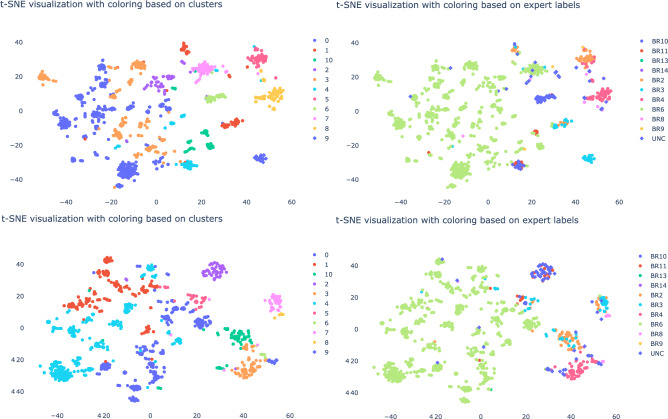
The t-sne visualization for company C obtained with finWalk (top) and DeepWalk (bottom), with on the left the clusters as indicated by the agglomerative clustering algorithm and on the right the expert’s labels.

Having defined the core processes of the companies, we will now move on to evaluate the obtained embeddings. For each node in the network we have a vector $x_i$ of length $k=8$. To plot each vector we reduce the 8 dimensions to 2 dimensions. We use the t-distributed stochastic neighbor embedding (t-sne) algorithm^33^ to obtain a 2-dimensional representation that preserves the cluster structure in the 8-dimensional representation. This enables us to visually inspect the quality of the clusters. In Figs. 3, 4 and 5 we plot the t-sne visualization of the vectors $x_i$ for each node. The left and right t-sne figures are equal except for their labels. In contrast, we show on the left the cluster’s labels and on the right the expert’s labels. We use agglomerative clustering to obtain the clusters. We cluster the vectors by minimizing the total variance within all clusters. That is, the sum of squares between the clusters’ centroid and its members for all clusters in Euclidean space. We start with singleton clusters; each vector $x_i$ is a cluster on its own. We then merge the pair of clusters that results in the smallest increase of the total variance. We repeat this until we have reached the desired number of clusters. In our case, the number of business processes identified by the expert. This process is referred to as agglomerative clustering with the Ward’s linkage algorithm^34^. From a visual inspection we observe that most clusters as indicated by the expert are also found by the clustering algorithm. Although some clusters lack completeness because the agglomerative clustering subdivided the population whereas the expert indicated this as one. This can be considered as the hierarchical organization of the business processes similar to the financial accounts. In addition to the visual inspection, we calculated the v-score. For company A, B and C we obtain a v-score of 0.71, 0.42 and 0.50 respectively. On the one hand, the v-score results of company A suggest that we can effectively capture the characteristics of a node. On the other hand, the v-score of 0.42 and 0.50 can partly be explained by the single large Sales (BR4) cluster for company B and the single large Purchases (BR6) cluster for company C whereas the clustering algorithm finds additional smaller clusters. When we force the algorithm to find fewer clusters for company B, we see an increase in the v-score from 0.42 to 0.50. Alternatively, we could also modify the v-score such that it puts more weight on the homogeneity. For company B and C, we bias the v-score towards homogeneity by setting the $\beta = 0.01$. As a result, we increase the v-score from (B) 0.42 to 0.76 and (C) 0.50 to 0.70. The clustering task performed well as a result of the learned vector representation that captures the essential characteristics of the nodes in the network. The agglomerative clustering algorithm can approximately reconstruct the same clusters as indicated by the expert. Therefore, we can state that we have a meaningful vector embedding that can be used for a variety of down-stream analyses, like clustering.

Moreover, we proposed a new random walk (see “Methods” section) to learn the vector embeddings. In order to demonstrate the need for this new random walk, we compared our finWalk strategy with the DeepWalk algorithm^21^. In Figs. 3, 4 and 5 we show the t-sne visualization for the finWalk (top) and the DeepWalk (bottom) strategy. Consequently, we can state that the clusters in the t-sne visualization of the finWalk are slightly more compact compared to DeepWalk. The v-score comparison between DeepWalk and finWalk results in 0.72 and 0.71 for company A, which suggests no strong preference between the two strategies. For company B, we have a v-score of 0.47 and 0.42, which suggests that DeepWalk is preferred. Lastly, for company C, we obtain 0.54 and 0.50, which suggests a preference for DeepWalk. Although both strategies result in good embeddings, in the simulated case we show superior results obtained with the finWalk strategy.

### Evidence of a useful flow network for predictive models

We find that the network structure extracted from transaction data has a positive effect on the prediction error of the predictive models.

We investigated what role the high-level flow network plays for the predictive models. Therefore, we propose two key categories of experiments, i.e. predictive models that do not use flow information and predictive models that do. For the latter we use expert’s flows and cluster’s flows. This gives us the following categories of models:

- *Account labels* (AM)—naive model where we ignore the in- and outflow and construct the net position of a financial account. In other words, when we have debit (inflow) and credit (outflow) entries, we aggregate them to obtain the net position at time *t*.
- *Credit-Debit labels* (CD)—naive model that operates only with in- and outflows, in other words, it does not use any information about the company’s accounting flow structure but only whether an account is debited or credited.
- *Expert’s labels*—the financial statements network flow with flow labels annotated by an expert (see Appendix A in Boersma et al.^15^). Thus, compared to the CD-model we add additional information of the process context, e.g. there can be an outflow (credit) of Revenue caused by a Sales and a separate outflow (credit) caused by another process. Here the process context is obtained by expert annotation.
- *Cluster’s labels*—similar to the Expert’s labels but here the process context is obtained by our network embedding and clustering algorithm.

For illustrative purposes, we picked three predictive models. Each model will be tested in accordance with the relevant model types. For each model we select all the transaction data that belongs to the business process. We constructed monthly summed totals for all models. For example, in the Revenue Credit/Debit-model we select all transactions that include a Revenue credit and all transactions that include a Cost of Sales debit. For this set, we construct monthly totals for Revenue (credit) and Cost of Sales (debit) by aggregating the amounts in the transactions. We calibrate the regression model in Eq. (1) with the obtained monthly totals. For the Revenue model with Expert’s labels and Cluster’s labels, we select a subset of the transactions related to the Sales process and the Goods dispatch process. We aggregate the Revenue (credit) amounts from the Sales process subset and the Cost of Sales (debit) amounts from the Goods dispatch process subset into monthly totals. We use the totals to calibrate the regression model in Eq. (1). Figure 1 shows the total monetary flows of the Revenue model labelled by the expert in red. Especially for the Cost of Sales financial account, it becomes clear that we select the subset of (debit) transactions from the Goods dispatch process. In a similar way, we can construct a flow network with our obtained clusters and use these as input for the predictive models. The section below describes the three models.

The first predictive model relates to the profit margin, i.e. the difference between the cost price of goods and the selling price. This results in the following relationship between flows:

1 $$\begin{aligned} \text {Revenue (credit)} = \alpha _0 + \alpha _1 \text {Cost of Sales (debit)} \end{aligned}$$

where $\alpha _0, \alpha _1$ are coefficients.

The second predictive model describes the purchases processes, where goods are bought and delivered. When goods are ordered, we observe an inflow of the inventory suspense account. When goods are delivered, we observe an outflow of the inventory suspense account. This results in the following relationships between flows:

2 $$\begin{aligned}&\text {Inventories suspense account (debit)} \\&\quad = \alpha _0 + \alpha _1 \text {Inventories suspense account (credit)} \end{aligned}$$

In the last model we predict the tax position. Here we study the changes in tax that are caused by selling goods, hence we expect a relationship between the Revenue position and the Tax position.

3 $$\begin{aligned} \begin{aligned} \text {Tax} = \alpha _0 + \alpha _1 \text {Revenue} \end{aligned} \end{aligned}$$

**Table 1:** This table shows the MAPE score for the three models of company A for the relationship based on the profit margin, the purchase and delivery of goods, and the tax payments related to sales. For the models that use the Cluster’s labels we added the standard deviation over 10 repetitions of the experiment. The lowest score is obtained by the Expert’s labels.

Model	Model revenue	Model inventory	Model tax
Account model	–	–	285.0
Credit/debit model	8.43	13.88	256.59
Expert’s label	3.41	13.88	9.31
Cluster’s label	$4.37 \pm 2$	$14.36 \pm 1$	$50.55 \pm 15$

To evaluate the predictive performance, we compare the MAPE score (see “Methods” section for more details and SI S4 for the regression tables). The MAPE score has an intuitive interpretation as the percentage of error and therefore can easily be compared between models. We calculate the MAPE score by calibrating the model based on 1 year of data and compute the MAPE score based on the predicted and true value. The calculation of the vectors $x_i$ is based on sampled paths in the network, therefore the vectors and resulting clusters can have small variations. To counter this effect, we repeated the experiment 10 times and report the average MAPE scores and the standard deviation. Table 1 shows the MAPE score for the three models and four setups. The scores of the Tax model suggest that using the business process context increases the predictability when multiple processes interact with a financial account. We observe a high MAPE score when we relate the net financial position to each other whereas the expert’s label and the Cluster’s label model show significantly lower MAPE scores. Nonetheless, the low $R^2$ value for both the Expert’s ($R^2 = 0.15$, see SI S4.3.3) and Cluster’s ($R^2 = 0.13$, see SI S4.3.4) labels suggests a weak linear relationship between the two variables for company A. In the case of the Inventory model, we do not expect a large difference between Expert’s labels, Cluster’s labels and the CD-labels because there is only one in- and outflow. The MAPE scores for all three types are therefore also approximately equal to each other. Furthermore, for the Revenue model there are multiple processes interacting with Revenue and Cost of Sales, but there is one major flow and several smaller flows. Therefore, we observe in Table 1 that both the Expert’s labels and Cluster’s labels outperform the simple in- and outflow model, although we still observe that the Expert’s label predictions outperform the Cluster’s label predictions. In general, the performance is not yet at par with an expert. This introduces a trade-off between time and accuracy: a high-level overview of the monetary flows can be obtained with relative ease.

#### Simulated data

To strengthen our validation we simulated, in addition to the expert validation, transaction data for which the ground truth is known. For the generated data, we use the SimPy discrete event simulation to simulate the company’s business process activities (see SI S3 for details). That means, we simulate, for example, a Sales process that eventually triggers the Collection process and we generate the corresponding transaction records. Each transaction record is labeled by the processes it originated from.

Before proceeding to examine the clusters, we discuss the simulated trade entity. The simulated trade entity has the following eight core processes: Collection, Depreciation, Fixed Assets, Goods delivery, Payroll, Purchase, Sales with 21% VAT and Sales with 6% VAT. From these generated transaction records, we obtained a network with 663 financial accounts and 5,551 business processes.

**Figure 6:**
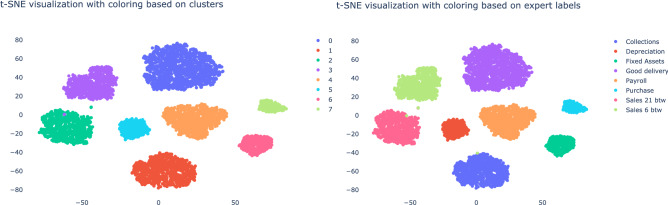
The t-sne visualization of the vector embeddings for the simulated data with the finWalk strategy. On the left you see the clusters as obtained by the agglomerative clustering algorithm and on the right you see the ground truth Cluster’s labels.

Let us now consider the clusters obtained from the vector embeddings. Figure 6 shows the t-sne visualization from the obtained vector embeddings. On the left we show the ground truth business process labels. On the right we show the eight clusters as obtained by the agglomerative clustering algorithm. The v-score of 0.98 indicates that our approach is able to find the clusters we generated to a high degree.

**Figure 7:**
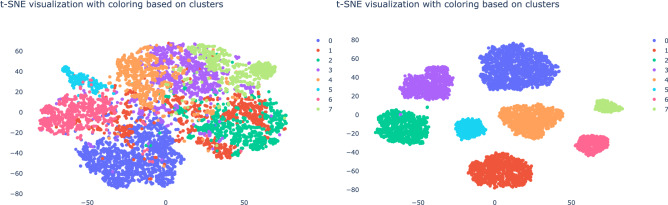
(Left) shows the t-sne visualization of the vector embedding obtained by a random walk for the simulated data. Right) shows the t-sne visualization of the vector embedding of the finWalk strategy. The images show that the finWalk strategy yields a better cluster formation.

Subsequently, we will turn to the experimental evidence of the finWalk strategy. Figure 7 shows that the DeepWalk gives a different embedding with a lower v-score of 0.55 than the finWalk strategy with 0.98. We observe that the DeepWalk strategy yields more dispersed clusters than for the finWalk strategy. The visualization and v-score strongly suggest that the finWalk strategy is better at capturing the essential characteristics of the business processes.

**Figure 8:**
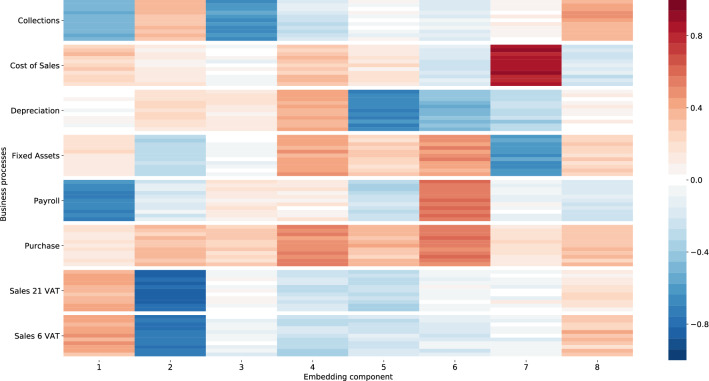
Visualization of vectors $x_i$ grouped in clusters. On the y-axis we display the name of the clusters and the x-axis shows the vector element with $k=8$. For each cluster we show 10 vectors $x_i$ that belong to that cluster. Vectors $x_i$ that belong to the same cluster are similar while, for example, the cluster Collections is different from the cluster Purchase.

**Figure 9:**
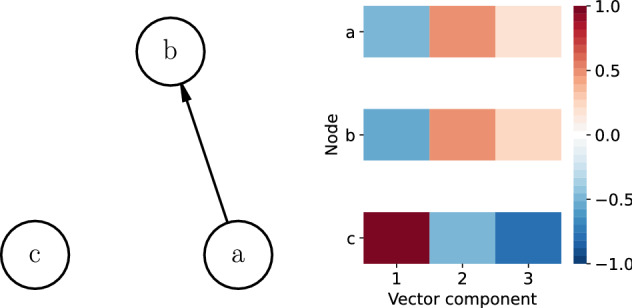
On the left an example network G. Node *a* and *b* are connected, therefore we expect that the vectors $x_a$ and $x_b$ of nodes *a* and *b* have values closer to each other compared to node *c*. On the right the visual representation of a $k=3$ dimensional vector for each node. We observe that the colors of node *c* are different from those of nodes *a* and *b*.

Moving on now to consider the embedding vectors of the business processes in more detail, we plot a heatmap of each vector grouped by their core process in Fig. 8. Each node is represented by an 8-dimensional vector $x_i$. We color the numbers of the vector: values close to 1 are red and values close to -1 are blue (see Fig.  9 for an example). This enables us to visually inspect the similarity between a set of vectors; if the colors are similar, then the vectors are similar. We select a couple of nodes that belong to the same business process cluster, for each node we visualize their vector. We do this for multiple business process clusters. Figure 8 shows 10 nodes for each business process, which confirms our expectations. We expect that nodes in the same business process group have a similar vector color. On the other hand, we expect that vectors from different business processes have different colors. In Fig. 8 we observe that the vectors from the same group have similar colors, while between the different groups the differences in color are more apparent. This illustrates that we effectively captured the core processes.

To assess the robustness of the approach we performed a sensitivity analysis (see SI S1). Here we used the One-Factor-at-a-time approach to test how the v-score is impacted for various parameter settings. We performed the sensitivity analysis for the $\lambda$ parameter (see “Methods” section), the window size, walks per node, embedding size and training steps (see Skip-Gram section). The results show that a $\lambda$ of 10 and a window size of 2 is preferred. Furthermore, for increasing training steps and walks per node we observe an increased v-score. For the embedding size, 8 seems to be the most appropriate. Larger embedding sizes decrease the v-score slightly.

## Discussion

The vector representation learned is a meaningful abstract encoding of the node in the financial statements network. Therefore, we can apply clustering algorithms using the vector representation that yields similar clusters as manually annotated by the expert. This annotated data can be used for down-stream tasks like the predictive relationships between financial accounts; for example, revenue, inventory and tax models.

However, we do notice a significant difference in v-score between the experiments with the simulated data and the real data. This is partly due to the expert’s Unclassified business processes; here the Unclassified business processes are spread between multiple clusters and therefore affect the homogeneity of the v-score in a negative way. When we exclude the Unclassified business processes from the evaluation, we observe a difference in v-score between $-0.05$ and 0.03. We analyzed the journal entries of the Unclassified category and found that the expert uses information from the source (sub-ledger or user) that generates the transactions to classify the business process. We found that many of the Unclassified business processes can be traced back to manual entries, or unexpected journals, while they are structurally equivalent to nearby business processes in the embedded space. This explanation only accounts for a small part of the difference between simulated and real data. In addition, we found that expert’s clusters are divided into smaller clusters by the agglomerative clustering. As a result, the v-score is lower because the completeness of these clusters is low. When we bias the v-score to the homogeneity, we observe increases in v-score between 0.10 and 0.34. Forcing the agglomerative clustering to find fewer clusters also results in a v-score increase. For the remaining part, we believe that the complexity of the real data is another plausible explanation. In the simulated data we attempted to mimic the characteristics of the journal entry data; improving this simulation might give v-score results that are closer to what we observe for the real data.

We have shown that our finWalk procedure outperforms the existing DeepWalk method for the simulated dataset. A reason for this is that the finWalk traverses back- and forward during the sampling steps and therefore explores the local neighborhood of the network better. Nonetheless, we did not find consistent results on the real dataset. We found that DeepWalk is similar in performance or outperforms finWalk on the real dataset. Domain-specific random walks could improve the embedding results^26, 28^, but this requires additional investigation and fine-tuning.

It is worth noting that some clusters are divided into multiple smaller clusters whereas the expert annotated these clusters as one. The label related to this cluster is the Purchase process, which is a more complex process compared to others. This indicates that some sort of hierarchical structure is learned in the embedded space similar to the hierarchical structure present in the financial account hierarchy. However, this introduces a trade-off. On the one hand, preserving all information yields the original network which is intractable for analysis purposes. On the other hand, a high cut-off point in combination with a small number of clusters results in a simple network with the risk of losing significant context information. Nonetheless, our results demonstrate the novelty of the embedding approach where we can split larger processes into smaller sub-processes.

For the predictive relationships we see that financial accounts that interact with multiple processes benefit from the network approach. Here flows are separated according to their business process context and these yields improved predictive performance while more simple processes, e.g. in- and outflow, do not show a significant improvement in predictive ability.

To conclude, we argue that structural information increases the predictive performance and that our method can effectively capture this structural information from real transaction data. The wiring diagram of the network contains a lot of valuable information. The position of a financial account within this network somehow encodes its function within this financial system. Moreover, algorithmic approaches in the audit could enhance the quality of the audit and reduce review findings by regulators such as the PCAOB. These data-driven methods could provide a consistent and objective way to analyze audit data and assist the auditor. In our specific example, the high-level monetary flows of an entity could assist the auditor in an initial risk assessment during an audit. And the predictive relationships could be used to detect inconsistencies. Moreover, there is active research to objectively quantify the amount of audit assurance obtained from predictive models^35^. For future work we recommend to investigate other predictive models and down-stream tasks that could benefit from the vector representation.

## Methods

### Financial statements network

In this section we introduce the financial statements network and how to extract this from transaction data^15^. A financial statements network is constructed from journal entry data, which describe the change in financial position and that is readily available in all companies. The journal entry records show how much money flows from one set of financial accounts to another set. These entries are generated by their underlying business process, for example, the Sales process. Table 2 shows journal entries for a Sales process and the Goods dispatch process. This data is used to construct a bipartite financial statements network $G = (A \cup B, E)$ with *A* as the set of financial account nodes and *B* as the set of business process nodes and $E \subseteq A \times B$ as the set of edges. The set of financial accounts can be obtained from the journal entry data. For example, from Table 2 the set of financial accounts would be all the unique names of all the journal entries. A business process is derived from the journal entry structure. The structure represents the relative amounts debited and credited for each financial account. Although amounts can be different, all journal entries with the same structure are considered equal. A formal definition of a business process can be written as^15^:

4 $$\begin{aligned} B : \sum ^m_{i=1} \alpha _{i} A_{i} \Rightarrow \sum ^n_{j=1} \beta _{j} A_{j} \end{aligned}$$

where *m* is the number of credited financial accounts, *n* is the number of debited financial accounts and $\alpha _i$ is the relative amount with respect to the total credited and $\beta _j$ the relative amount with respect to the total debited. The edges between nodes $A_i$ or $A_j$ and *B* are the coefficients $\alpha _i$ and $\beta _j$ from the business process definition in Equation 4.

### Node embedding

The networks can be quite large, therefore we propose a method to reduce the dimension of the financial statements network *G*. To reduce the size of the network, we group nodes that are similar. We calculate the similarity between nodes with the network embedding technique. For each node in the network we calculate a $k-$dimensional vector $x_i$ that encodes its characteristics. Figure 9 shows an example network, on the left we see the network and on the right we see the vector $x_i$ for each node in the network. We use the Skip-Gram algorithm to find the vector values of each node, such that, when two nodes $B_i$ and $B_j$ are similar, then the distance between their corresponding vectors $x_i$ and $x_j$ should be close in Euclidean space or have a high cosine similarity.

**Table 2:** Transaction with ID 1 is a Sales transaction and transaction with ID 2 is a Goods dispatch transaction.

ID	Name	Journal	Date	Debit	Credit
1	Trade Receivables	Sales ledger	1-1-2019	2420	
1	Revenue	Sales ledger	1-1-2019		2000
1	Tax	Sales ledger	1-1-2019		420
2	Cost of Sales	Journal ledger	2-1-2019	1500	
2	Inventories	Journal ledger	2-1-2019		1500

#### FSN random walk

Perozzi et al.^21^ demonstrated that these vectors can be learned by generating random-walk samples from the network. To generate a random walk from the network, the DeepWalk implementation uses the following transition probability to select the next *target* node when we are in the *source* node:

5 $$\begin{aligned} p(target | source) = \frac{1}{N(source)}, \end{aligned}$$

where *N*(*source*) is the number of neighbors of the *source* node regardless of the edge direction, i.e. we select a new *target* node uniformly from all neighbors of the *source* node. Follow-up research shows that various random-walk strategies have an impact on the learned embeddings^26, 28^.

We propose a new random-walk strategy for our specific clustering task at hand. The intuition behind the finWalk random-walk strategy is as follows. We consider the random walk to be a walk through a local structure of the network. The network sampling procedure has two types of transitions: forward step – selecting edges proportional to their edge weight – and a backward step – selecting edges that have the same direction and are similar in weight. We start at a selected business process node and take a single step forward, i.e. we follow the edge direction with the forward transition probability; Next, we select an edge that is comparable in weight and direction to the edge we arrived from. We use the backward transition probability to sample a similar edge. Then, we walk from a business process node to a financial account node with the forward transition probability. For the next step, we select an edge that is alike in weight and direction to the edge we arrived from with the backward transition probability to arrive in a business process node. We repeat this until we have reached the desired length of the random walk. We iterate between the forward and backward transition probabilities because the forward transition probabilities enable us to explore new parts of the network, while the backward transition probabilities bias to edges that are similar to what we have already explored. For example, a Sales transaction could have various Tax rates, e.g. 21% and 6%; when we walk from a business process node to a financial account node via the 21% tax edge, then for the walk backwards, we want to select another business process with almost the same tax rate. By making the random walk biased, we generate sequences of samples where business processes with similar characteristics share the same path.

#### Forward transition

For the forward transition probability we select the edge proportional to its edge weight. Let *W* be the set of edge weights of all reachable target nodes from the source node. Then the forward transition probability to a target node with edge weight $w_t$ is:

6 $$\begin{aligned} P(target | source) = \frac{w_t}{\sum _{w_i \in W} w_i} \end{aligned}$$

where we aggregate all the weights $w_i$ of all targets reachable from the source node.

#### Backward transition

 Assume that we have a source financial account node *A* and we arrived at the source node via the edge with weight $w_s$. Let $w_t$ be the weight of the edge of the target node, then the transition probability is determined by:

7 $$\begin{aligned} P(target | source) = \sigma (( 1 - |w_s - w_t|) \times \lambda ) \end{aligned}$$

where $|w_s - w_t|$ is the absolute difference between the two edge weights and $\sigma : {\mathbb {R}}^k \rightarrow [0,1]$ is a soft-max mapping of a vector to a probability distribution. Let $z = (z_1,..,z_k)$ be the vector of values, then $\sigma$ is defined as:

8 $$\begin{aligned} \sigma (z) = \frac{exp(z)}{\sum ^k_{i=1}exp(z_i)} \end{aligned}$$

and $\lambda$ is a scalar to bias the transition to edges $w_t$ that are closer to $w_s$.

### V-score

The v-measure^32^ is the harmonic mean of the homogeneity score and the completeness score. The score consists of two elements, the classes *C* and the clusters *K*. The contingency table represents the number of data points that are elements of a cluster *K* and class *C*, the element $a_{ij}$ represents the number of data points that are part of cluster $k_j$ and class $c_i$. In the equations below we refer to *N* as the number of data points and |*C*| as the number of classes and |K| as the number of clusters.

#### Homogeneity

The homogeneity is calculated as the conditional entropy of a class distribution for a given clustering. A cluster is homogeneous if all members of that cluster belong to a single class. Clusters with multiple classes are not homogeneous.

9 $$\begin{aligned} h = {\left\{ \begin{array}{ll} 1 \quad &{} \text {if H(C, K) = 0}\\ 1 - \frac{H(C | K)}{H(C)} \quad &{} \text {else} \end{array}\right. } \end{aligned}$$

where

10 $$\begin{aligned} H(C | K)= & {} - \sum ^{|K|}_{k=1}\sum ^{|C|}_{c=1} \frac{a_{ck}}{N} log \frac{a_{ck}}{\sum ^{|C|}_{c=1} a_{ck}} \end{aligned}$$

11 $$\begin{aligned} H(C)= & {} - \sum ^{|C|}_{c=1} \frac{\sum ^{|K|}_{k=1} a_{ck}}{|C|} log \frac{\sum ^{|K|}_{k=1} a_{ck} }{|C|} \end{aligned}$$

#### Completeness

completeness measure is the conditional entropy measure of the completeness of a cluster. A cluster is complete when all members of a class belong to a single cluster. If the class is split into multiple smaller clusters, then the class is incomplete.

12 $$\begin{aligned} c = {\left\{ \begin{array}{ll} 1 \quad &{} \text {if H(C, K) = 0}\\ 1 - \frac{H(K | C)}{H(K)} \quad &{} \text {else} \end{array}\right. } \end{aligned}$$

where

13 $$\begin{aligned} H(K | C)= & {} - \sum ^{|C|}_{c=1}\sum ^{|K|}_{k=1} \frac{a_{ck}}{N} log \frac{a_{ck}}{\sum ^{|K|}_{k=1} a_{ck}} \end{aligned}$$

14 $$\begin{aligned} H(K)= & {} - \sum ^{|K|}_{k=1} \frac{\sum ^{|C|}_{c=1} a_{ck}}{|C|} log \frac{\sum ^{|C|}_{c=1} a_{ck} }{|C|} \end{aligned}$$

#### Harmonic mean

The v-score is the harmonic mean of the completeness and the homogeneity measures. A high v-score shows that all classes belong to a single cluster, a low v-score shows that the clusters consist of mixed classes or that a class is subdivided into multiple clusters. The v-score is formally defined as

15 $$\begin{aligned} v = (1 + \beta ) \frac{h * c}{\beta * h + c} \end{aligned}$$

where $\beta$ determines the weight between completeness and homogeneity. A $\beta$ higher than 1 results in a higher weight for completeness and vice versa.

### Mean absolute percentage error

The Mean Absolute Percentage Error (MAPE)^36^ is a scale-independent measure that enables us to compare performance between the different types of models. The MAPE value is the absolute value of the sum of the differences between the predicted value ${{\hat{y}}}_i$ and the observed value $y_i$ divided by the observed value $y_i$. The MAPE value is defined as:

$$\begin{aligned} \text {MAPE} = \frac{1}{N}\sum ^N_{i=1}\bigg | \frac{y_i - {{\hat{y}}}_i}{y_i} \bigg |, \end{aligned}$$

where ${{\hat{y}}}_i$ is the predicted value, $y_i$ the observed value and *N* the total number of datapoints.

**Figure 10:**
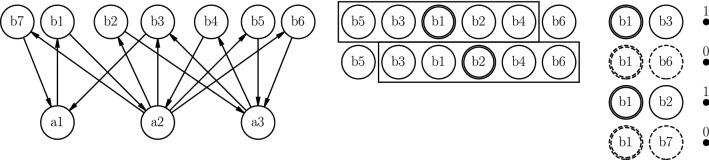
(Left) the example network with b1-b7 as business process nodes and a1-a3 as financial account nodes, we sample a path from this network with DeepWalk or finWalk. Middle) one sampled path with a sliding window at the first and the second position. The context box indicates the full window with *b*1 and *b*2 as target nodes. Right) generated training pairs from the first window for the prediction model, solid lines are examples from the sampled path (positive items) and dashed pairs are negative items.

### Skip-gram

We use the Skip-Gram model as proposed in Mikolov et al^24^. The Skip-Gram model was originally developed to learn statistical relationships in language between a **target** word and its *context*. For example, *the* **cat/dog** *is eating the food*. Inferring these relationships from a large text corpus enables us to learn similarities between words. Target words that can be used interchangeably in the same context are similar, like cat and dog in the example sentence. Expressing the similarity between the words cat and dog is non-trivial, as they are both pets and therefore related. Skip-Gram enables us to replace the words by their learned vector representation of real values, such that, words that have similar meaning have vector representations that are close to each other in the vector space. The vector representations are learned from a large text corpus by turning it into a prediction problem, where we predict the context given the target word. The learned vectors minimize the prediction error for all training sentences from the text corpus. Therefore, the problem of similarity between words like cat and dog can be expressed as the Euclidean distance or cosine distance between their respective vector representations.

These embeddings can also be used outside the context of statistical language modelling, such as in the case of networks. Here the objective is to learn the similarity between nodes. However, unlike a text corpus, we do not have a sequence of nodes which corresponds to the notion of a sentence in a network. Perozzi et al.^21^ showed that we can generate a sequence of nodes with a random-walk on the network (DeepWalk). These sampled paths of nodes are used in the same way as the sentences in the language model. Therefore, nodes in a network are similar when the distance between their vector representation is small.

For the financial statements network, we sample the network with finWalk or DeepWalk to obtain a path. Because we learn the statistical relationship between a target and the context in a sequence of nodes, changing between DeepWalk and finWalk results in different learned vector representations. Intuitively, these random-walks simulate the flow of money in the financial system, especially, because we derived the network structure from real transactions. DeepWalk is a random-walk on the undirected and unweighted version of the financial statements network and finWalk is a random-walk that uses both the weights and direction of the edge. In the generated path, we ignore financial account nodes in the path because we are interested in the similarity between business process nodes.

To learn the vector embeddings, we assume that we sampled a path of nodes with either finWalk or DeepWalk. For example, we sampled the following sequence of nodes {b5, b3, b1, b2, b4, b6} from the example network in Fig. 10 (left). From the resulting path, see Fig. 10 (middle), we generate training pairs. We select **b1** as the target node, {[*b5, b3, ***b1**, *b2, b4*], b6} and select all nodes for a window size of 2. Because we use negative sampling in the Skip-Gram model, we generate positive items and negative items. Figure 10 (right) shows the generated positive and negative training pairs. The positive items come from the sampled path, e.g. [(b1, b5), 1], [(b1, b3), 1], [(b1, b2), 1], [(b1, b4), 1], and the negative items are generated by sampling a random node from the network, e.g. [(b1, b6), 0], [(b1, b7), 0]. The positive and negative training pairs are used to learn to predict whether nodes are neighbors (positive pair) or not (negative pair). We use a sliding window over the sampled paths to generate all training pairs. We use the soft-max function to calculate the prediction probabilities from the vector representations of each node for all training pairs. To minimize the error between the predicted output and the known output (0/1), we optimize the vector representation of each node. We use the Adam-optimizer^37^ to compute the optimal vector values. For the Skip-Gram model we use an 8-dimensional vector representation for each node, a batch size of 256 and 100,000 training steps and 512 negative samples. To generate the sample paths, we use a window size of 2; 30 walks per node, walk length of 10 and a $\lambda$ parameter of 10.

## Supplementary information

Supplementary Information.
