# Supplementary material for: Reducing the complexity of financial networks using network embeddings
Source: Sci Rep. 2020 Oct 12;10:17045. doi: 10.1038/s41598-020-74010-2 (PMC7550348; doi:10.1038/s41598-020-74010-2)
Supplement: Supplementary file 1 — Supplementary Information. [file 41598_2020_74010_MOESM1_ESM.pdf]

Reducing the Complexity of Financial Networks using Network Embeddings

M. Boersma[1,2], A. Maliutin[1,2], S. Sourabh[1], L.A. Hoogduin[3], and D. Kandhai[1]

[1]Computational Science Lab, University of Amsterdam, Amsterdam, the Netherlands
[2]KPMG, Amstelveen, the Netherlands
[3]KPMG Global Solutions Group, Berlin, Germany
[*]m.boersma@uva.nl

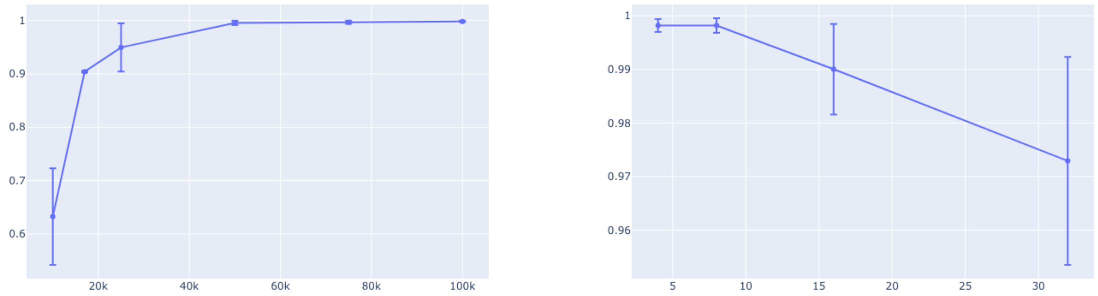

Figure S1. Left) v-score as a function of the number of training steps in the Skip-Gram model. Right) v-score as a function of the embedding size.

Contents

S1 Sensitivity analysis **S2**
S2 Data **S4**
S3 Simulated data **S4**
S4 Regression tables **S6**
S4.1 Revenue model S6
Credit-Debit label • Expert's label • Cluster's label
S4.2 Inventory model S7
Credit-Debit label • Expert's label • Cluster's label
S4.3 Tax model S8
Account label • Credit-Debit label • Expert's label • Cluster's label
References **S8**

Supplementary materials

S1 Sensitivity analysis

We investigated the sensitivity of the v-score as a function of the input parameters. The sensitivity can be categorized in two main components, the Skip-Gram model[1] and the random-walk strategy. We use two datasets, the simulated data and the real data of a company A to assess the change in v-score for certain parameter settings. For the sensitivity analysis we use a One-factor-at-a-time approach where we change one parameter and fix all others.

Before we proceed to discuss the sensitivities, we first explain which parameters we included in the experiment. For the Skip-Gram model we investigate two parameters: the training steps and the embedding size. For the random-walk strategy we investigate the following parameters: λ, the number of walks per node, the window size and the number of steps we take in a single walk. We repeat each experiment four times and determine the median and error bars as the standard deviation of the v-score.

Now moving on to the sensitivity results, we first show the results of the simulated data and then discuss the results of the real data. Figure S1 shows the results for the training steps and the embedding size for the Skip-Gram model. The results suggest that the v-score converges when we increase the number of training steps. Furthermore, a preferred embedding size of 8 is implied by the second plot. This is in line with the suggested[2] embedding size that is the 4th root of the number of unique nodes. Let us now consider the random-walk parameters; Figure S2 shows the v-score for various parameters of the random walk. The λ plot implies a preference for higher λ values, this can be explained by the Sales process with 21% VAT and 6% VAT. A higher λ enforces a selection bias toward similar edges. As a result, the groups can be distinguished. For the walks per node and steps per walk we do not see any strong preference. Any window size between 1-4 provides highly accurate results whereas a window size of 5 shows a significant drop in v-score.

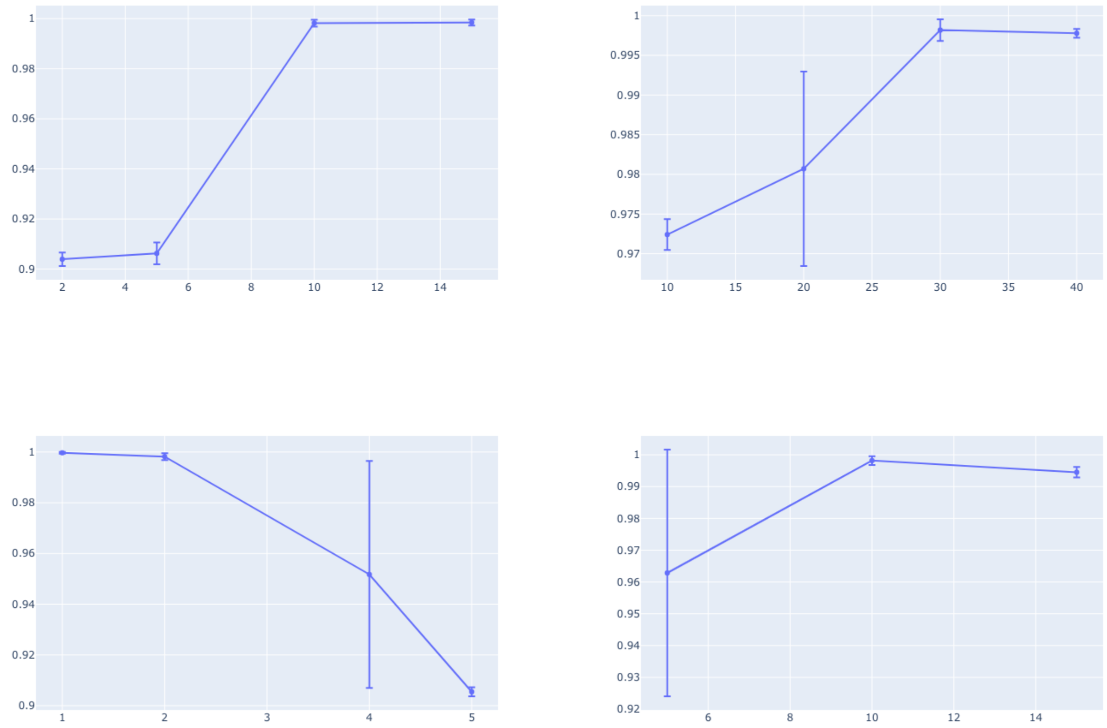

Figure S2. Top left) v-score as a function of the λ. Top right) v-score as a function of the walks per node. Bottom left) v-score as a function of the window size. Bottom right) v-score as a function of the steps per walk.

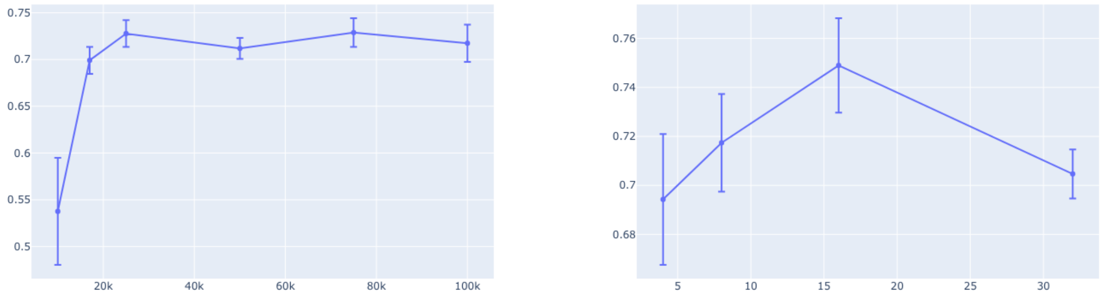

Figure S3. Left) v-score as a function of the number of training steps in the Skip-Gram model. Right) v-score as a function of the embedding size.

Having studied the results from the simulated data, we will now show the sensitivity analysis for the real data. Figure S3 shows results for the training steps and embedding size of the Skip-Gram model. In line with the results obtained from the simulated data, we observe that increasing the training steps increases the results. For the embedding size we see a preference for a larger embedding size of 16, whereas we would expect a similar embedding size based on the number of nodes. Turning now to the parameters of the random walk: Figure S4 shows that there is a preference of a low λ. In the real data we have more business processes with only a single input and output edge. As a consequence, the λ parameter has no impact because it requires at least two edges. Figure S4 bottom left suggests that higher window sizes are preferred although the difference is only small. A possible explanation is the number of one-to-one connections, therefore the walk has to traverse further to learn the relationships. In addition, the right bottom plot suggests that 10 steps per walk is preferred.

Thus far, we have shown the change in v-score for the parameters as listed above in two datasets. For almost all parameter settings we obtain good results, suggesting that our approach is robust. Despite this, for some parameters we see a slight preference for specific settings, which we explained above.

S2 Data

We used the journal entry data of three mid-size companies from the Netherlands. We used the data from 2014, 2015 and 2019 for our analysis. One data set is approximately 1,600 journal entries, the other set approximately 10,000 journal entries and the last set roughly 50,000 journal entries.

S3 Simulated data

In order to generate realistic transaction data, we use a discrete event simulation framework[3]. A company consists of a set of business processes, e.g. selling products, paying personnel, and some business activities generate journal entries that describe the change in financial position. In the discrete event simulation we simulate the activity of each business process. For example, we simulate that we sold an item and with a random delay the payment of the corresponding invoice. We defined the following processes; a schematic version is displayed in Figure S5:

- Collections: this process relates to the collection of money after selling goods
- Depreciation: depreciation of assets
- Fixed Assets: purchase of new assets
- Goods delivery: delivery of goods
- Payroll: employee benefits costs
- Purchase: purchases of, for example, office materials
- Sales 21 VAT: selling products with 21% tax

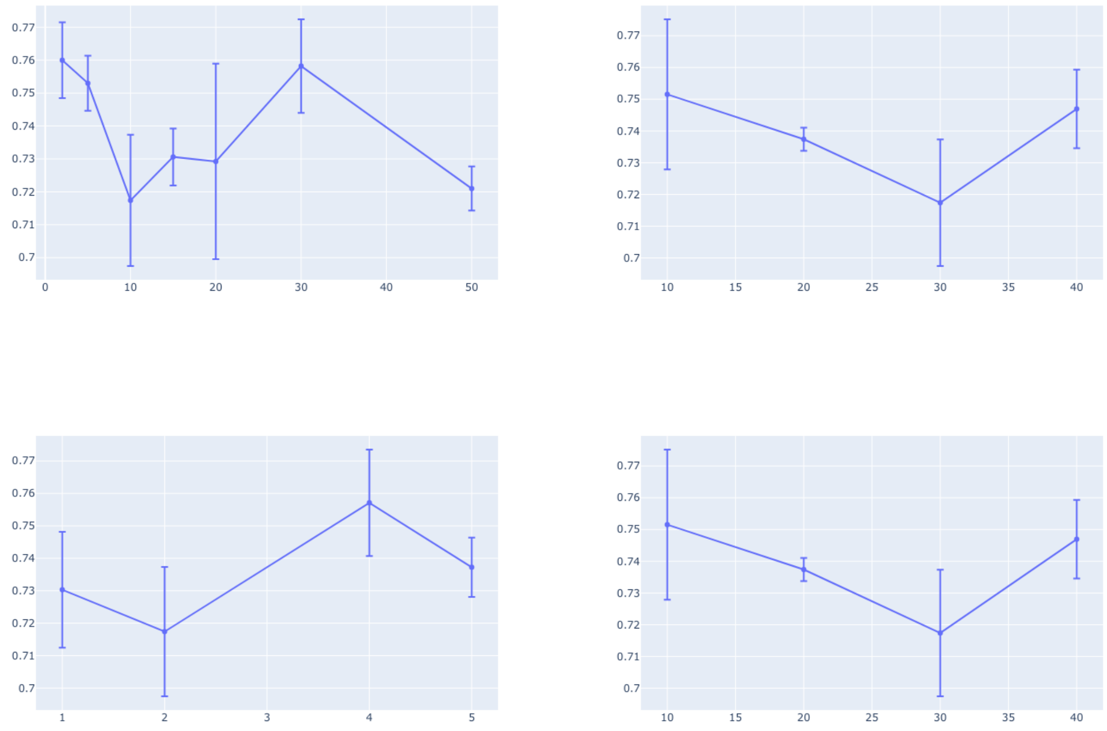

Figure S4. Top left) v-score as a function of the λ. Top right) v-score as a function of the walks per node. Bottom left) v-score as a function of the window size. Bottom right) v-score as a function of the steps per walk.

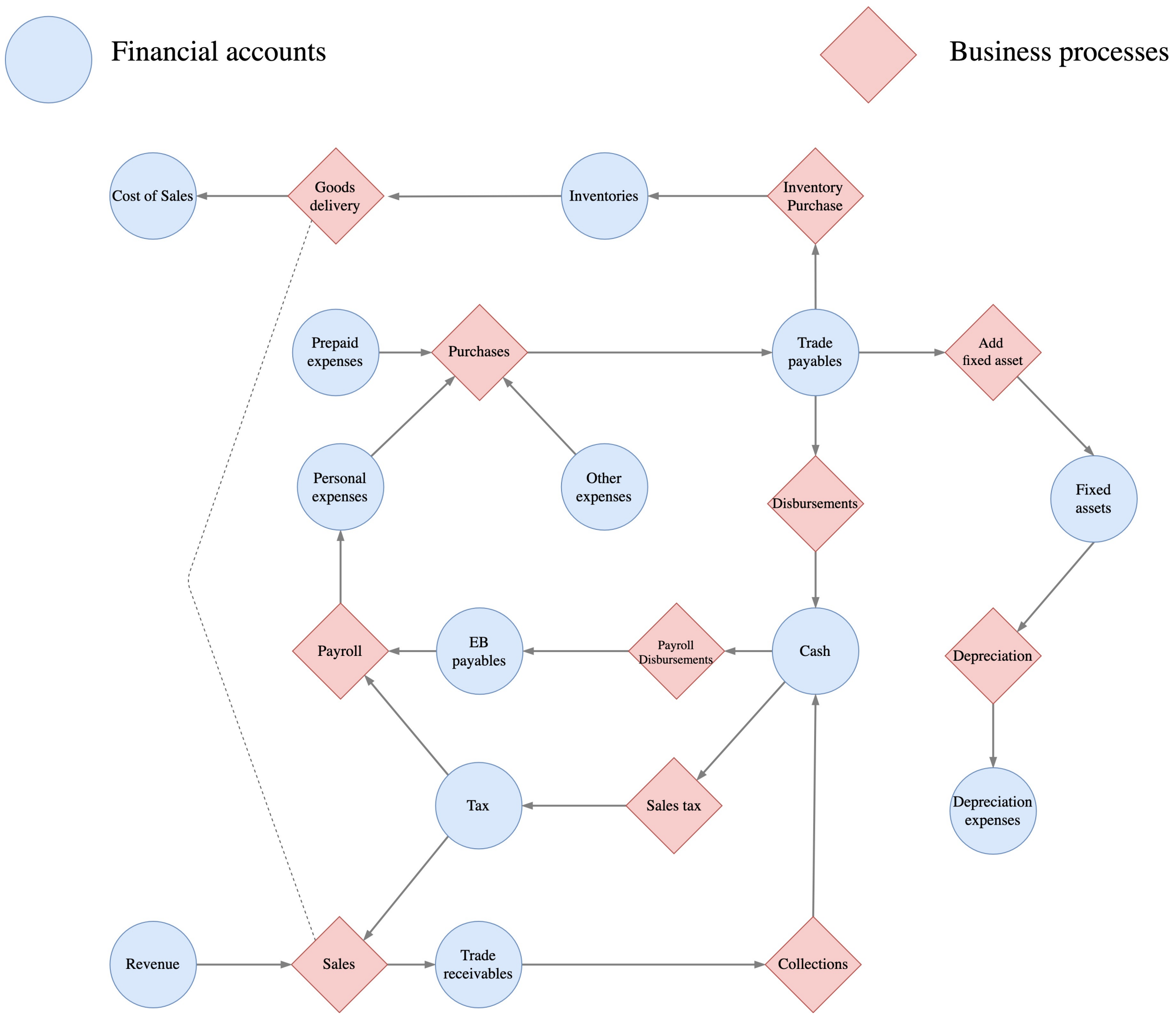

Figure S5. A schematic overview of the simulated processes. The edges represent debit (incoming edge) and credit (outgoing edge) bookings. The red squares represent the main business processes and the round circles the financial accounts.

- Sales 6 VAT: selling products with 6% tax

However, we noticed that in the real data the journal entries do not always adhere to the accounting standards, which suggest simple journal entries to describe the change in financial position. In practice, we see journal entries that have many more financial accounts in the journal entry than we would expect based on the simple process description. Therefore, we propose to enhance our simulation by generating more realistic journal entries which have additional financial accounts in addition to the standard financial accounts. To this end, we add additional financial accounts to our simulation. We use the additional financial accounts when we generate a journal entry. For example, for a sales event we create a basic journal entry based on accounting knowledge but we add such additional accounts that move smaller amounts of value in the journal entry. As a result, we obtain journal entries that look more similar to entries in the real dataset. In our simulation framework we can generate networks of a particular size and thereby create networks that have realistic characteristics.

S4 Regression tables

For 3 predictive models and for 3-4 scenarios, we further specified the regression tables. From the 10 runs for the Cluster's labels we selected a single regression table and report the MAPE score of that run.

S4.1 Revenue model

The model:

$$\text{Revenue (credit)} = \alpha_0 + \alpha_1 \text{Cost of Sales (debit)} \quad (1)$$

where α_0, α_1 are coefficients.

S4.1.1 Credit-Debit label

	**R-squared:**		0.059	
	**Adj. R-squared:**		-0.034	
	**F-statistic:**		0.63	
	**coef**	**std err**	**t**	**P-value**
**const**	784873.74	176217.94	4.45	0.000972
**x**	0.197	0.041	4.77	0.000585

S4.1.2 Expert's label

	**R-squared:**		0.39	
	**Adj. R-squared:**		0.33	
	**F-statistic:**		6.33	
	**coef**	**std err**	**t**	**P-value**
**const**	653822.66	80889.39	8.08	0.000006
**x**	0.376	0.021	18.23	0.000000

S4.1.3 Cluster's label

We selected the regression table for a run with MAPE score 3.91.

	**R-squared:**		0.57	
	**Adj. R-squared:**		0.52	
	**F-statistic:**		13.33	
	**coef**	**std err**	**t**	**P-value**
**const**	126037.34	183558.54	0.68	0.506
**x**	1.83	0.028	64.85	0.000

S4.2 Inventory model

The model:

$$\text{Inventories suspense account (debit)} = \alpha_0 + \alpha_1 \text{Inventories suspense account (credit)} \quad (2)$$

where α_0, α_1 are coefficients.

S4.2.1 Credit-Debit label

	**R-squared:**		0.89	
	**Adj. R-squared:**		0.88	
	**F-statistic:**		81.41	
	**coef**	**std err**	**t**	**P-value**
**const**	31231.36	66407.90	0.47	0.647
**x**	0.945	0.041	23.14	0.000

S4.2.2 Expert's label

	**R-squared:**		0.89	
	**Adj. R-squared:**		0.88	
	**F-statistic:**		84.49	
	**coef**	**std err**	**t**	**P-value**
**const**	31710.76	65475.19	0.48	0.637
**x**	0.949	0.0402	23.58	0.000

S4.2.3 Cluster's label
We selected the regression table for a run with MAPE score 14.53.

	**R-squared:**		0.89	
	**Adj. R-squared:**		0.88	
	**F-statistic:**		81.41	
	**coef**	**std err**	**t**	**P-value**
**const**	31231.37	66407.90	0.47	0.647
**x**	0.945	0.041	23.14	0.000

S4.3 Tax model
The model:

$$\text{Tax} = \alpha_0 + \alpha_1 \text{Revenue} \quad (3)$$

where α_0, α_1 are coefficients.

S4.3.1 Account label

	**R-squared:**		0.094	
	**Adj. R-squared:**		0.0033	
	**F-statistic:**		1.04	
	**coef**	**std err**	**t**	**P-value**
**const**	443957.18	382669.31	1.16	0.27054
**x**	0.504	0.026	19.3	0.0000

S4.3.2 Credit-Debit label

	**R-squared:**		0.077	
	**Adj. R-squared:**		-0.015	
	**F-statistic:**		0.83	
	**coef**	**std err**	**t**	**P-value**
**const**	2.368720e+06	1.970231e+06	1.2	0.2545
**x**	-2.088	2.545850e-01	-8.20	0.000005

S4.3.3 Expert's label

	**R-squared:**		0.154	
	**Adj. R-squared:**		0.070	
	**F-statistic:**		1.83	
	**coef**	**std err**	**t**	**P-value**
**const**	52867.20	16115.60	3.28	0.007327
**x**	-0.027	0.00114	-24.01	0.000

S4.3.4 Cluster's label
We selected the regression table for a run with MAPE score 25.028.

	**R-squared:**		0.13	
	**Adj. R-squared:**		0.043	
	**F-statistic:**		1.50	
	**coef**	**std err**	**t**	**P-value**
**const**	-40532.32	69773.39	-0.58	0.573008
**x**	0.1087	0.0056	19.28	0.0000

References

1. Mikolov, T., Chen, K., Corrado, G. & Dean, J. Efficient estimation of word representations in vector space. *arXiv preprint arXiv:1301.3781* (2013).
2. Google. *Google Developers Blog* (2019 (accessed December 5, 2019)).
3. Simpy, T. Simpy 3. Accessed: 5-12-2019.
